# Exploring improvements in patient logistics in Dutch hospitals with a survey

**DOI:** 10.1186/1472-6963-12-232

**Published:** 2012-08-01

**Authors:** Wineke AM van Lent, E Marloes Sanders, Wim H van Harten

**Affiliations:** 1 Division of Psychosocial Research and Epidemiology, Netherlands Cancer Institute - Antoni van Leeuwenhoek Hospital, PO Box 90203, Amsterdam, BE 1006, The Netherlands; 2 Division of Psychosocial Research and Epidemiology, Netherlands Cancer Institute Antoni van Leeuwenhoek Hospital, PO Box 90203 Amsterdam, The Netherlands; 3 Department of Health Technology Services Research School of Management and Governance, University of Twente, Enschede, The Netherlands; 4 Present affiliation: IZIT, Enschede, The Netherlands

**Keywords:** Efficiency, Organizational, Organizational Management, Organization and Administration, Patient logistics, Operations Management

## Abstract

**Background:**

Research showed that promising approaches such as benchmarking, operations research, lean management and six sigma, could be adopted to improve patient logistics in healthcare. To our knowledge, little research has been conducted to obtain an overview on the use, combination and effects of approaches to improve patient logistics in hospitals. We therefore examined the approaches and tools used to improve patient logistics in Dutch hospitals, the reported effects of these approaches on performance, the applied support structure and the methods used to evaluate the effects.

**Methods:**

A survey among experts on patient logistics in 94 Dutch hospitals. The survey data were analysed using cross tables.

**Results:**

Forty-eight percent of all hospitals participated. Ninety-eight percent reported to have used multiple approaches, 39% of them used five or more approaches. Care pathways were the preferred approach by 43% of the hospitals, followed by business process re-engineering and lean six sigma (both 13%). Flowcharts were the most commonly used tool, they were used on a regular basis by 94% of the hospitals. Less than 10% of the hospitals used data envelopment analysis and critical path analysis on a regular basis. Most hospitals (68%) relied on external support for process analyses and education on patient logistics, only 24% had permanent internal training programs on patient logistics. Approximately 50% of the hospitals that evaluated the effects of approaches on efficiency, throughput times and financial results, reported that they had accomplished their goals. Goal accomplishment in general hospitals ranged from 63% to 67%, in academic teaching hospitals from 0% to 50%, and in teaching hospitals from 25% to 44%. More than 86% performed an evaluation, 53% performed a post-intervention measurement.

**Conclusions:**

Patient logistics appeared to be a rather new subject as most hospitals had not selected a single approach, they relied on external support and they did not have permanent training programs. Hospitals used a combination of approaches and tools, about half of the hospitals reported goal accomplishment and no approach seemed to outperform the others. To make improvement efforts more successful, research should be conducted into the selection and application of approaches, their contingency factors, and goal-setting procedures.

## Background

In the Netherlands, as in other Western countries, healthcare costs are rising [1]. To tackle this, the government has recently introduced market-based approaches aimed at limiting expenditure and improving the quality of healthcare [2]. Hospitals are now expected to improve the efficiency of their processes – all at a time when demand is increasing and the recruitment of sufficient staff is difficult. Simultaneously, the focus of quality management in healthcare is gradually shifting away from clinical effectiveness [3], to a greater emphasis on the organization of processes [4]. This focus is also acknowledged by the Institute of Medicine (IoM) that has declared that timeliness and efficiency are important aspects of quality in healthcare [5].

The challenges now faced by hospitals with regard to patient logistics can be compared with those faced by manufacturing organizations with regard to operations management (OM). OM research covers *“the activity of managing the resources which are devoted to the production and delivery of products and services”*[6]. Vissers and Beech defined health OM as: “*the analysis, design, planning, and control of all of the steps necessary to provide a service for a client*[7].” In the Netherlands, patient logistics is often used to describe health OM. Patient logistics was used in a report on logistics in healthcare [8] that was part of a national action programme to improve the quality and efficiency of Dutch hospitals from 2004 to 2008 [9]. According to this report patient logistics consists of *“the total path that a patient follows in the healthcare system as the result of a specific health question*[8]*.”* We conclude that patient logistics can be regarded as an element of the aspects efficiency and timeliness in the IOM definition of quality.

Historically, OM approaches in businesses gradually have started to overlap with quality management, for example in lean management and six sigma (for definitions, see additional file 1, page 2–3). Research showed that promising approaches such as benchmarking, operations research, lean management and six sigma, can be adopted in healthcare [10-14]. However, concrete evidence is lacking whether these approaches developed especially for the manufacturing sector can also be used in healthcare [15].

Despite such differences, reviews of specific approaches show that some of the approaches used by hospitals to improve patient logistics were derived from the manufacturing industry. Reviews on various of these approaches such as operations research models, [12], simulation [16], six sigma and lean management, [13] and business process re-engineering, [17] reported improved patient logistics in hospitals or recommendations for these improvements. These reviews concluded that the study designs adopted to evaluate the effects of the interventions were not always rigorous; most evaluations consisted of a pre-post analysis within a single organization, [13] and controls were often lacking. Not only have not these reviews featured hardly any papers with negative results, they have not provided insight into the selection and combination of approaches used in the hospital sector.

To our knowledge, little research has been conducted to obtain an overview on the use, combination and effects of approaches to improve patient logistics in hospitals. One exception has been Yasin et. al. [18], who surveyed the extent to which the following approaches were implemented in 108 Tennessee (USA) hospitals in 2002: continuous improvement (CI), total quality management (TQM), business process re-engineering (BPR), just-in-time techniques (JIT), organizational restructuring, job re-engineering (JR) and benchmarking (BM). Self- reported surveys found a 100% implementation rate for CI in for-profit hospitals and 98.7% in non-profit hospitals. TQM and BM scored above 60% in both hospitals types. BPR, JIT, JR and organizational restructuring were rated between 29.8% and 60%. Self-reported success was rated from very ineffective to very effective. The lowest success rate was reported for BPR in non-profit hospitals (68.2%), the highest for CI in for-profit hospitals (100%). In the same period, Sluijs et al. [19] examined the activities undertaken as part of quality management systems in Dutch medical institutions. The uptake seemed considerably lower than the implementation rate reported by Yasin et al. [18]. Either the methodologies were incomparable or the implementation rate was actually higher in the US hospitals.

Based on the above, we concluded that additional information was needed on approaches to patient logistics and on how they affect performance. In all 94 Dutch hospitals we therefore surveyed 1) the use of business approaches, 2) the tools used to support these approaches, 3) the reported effects of these approaches on performance, 4) the support structure that was applied, and 5) the methods used to evaluate the effects of the approaches.

## Methods

We used a survey whose content validity had been verified by three independent researchers and two consultants active in the field of patient logistics. All provided feedback on the research design and the survey.

After minor modifications, four respondents at four different Dutch hospitals piloted the survey. This led to textual modifications.

The survey consisted of five sections:

1. Hospital type. Three hospital types were distinguished: general hospital, non-academic teaching hospitals (not affiliated to universities), and academic teaching hospitals (affiliated to universities).

2. Approaches used to improve patient logistics. This section consisted of two questions, one focusing on approaches that had been used during the previous two years, and one focusing on the approach that had been used most intensively during the same period. We selected 11 approaches on the basis of 1) literature on the application of approaches to improving patient logistics in healthcare (see Additional file 1: survey) and, 2) the authors’ expertise and the validation of five independent experts. The table with the approaches also presents the literature upon which their selection has been based.

3. The frequency with which hospitals used tools or activities related to a specific approach to improve patient logistics (in the rest of this paper this will be called tools). We added this section after consulting the independent experts. As different hospitals might use different definitions for approaches of the same name, our intention was to provide greater insight into the tools that hospitals actually use to improve patient logistics. We asked respondents to rate the intensity at which their hospital used specific tools on a five-point Likert scale (from rarely used to almost always used). We excluded tools, such as are failure mode and effects analysis (FMEA) and poka yoke, that are intended primarily to bring about culture change or safety improvement. Although these tools indirectly contribute to the efficiency because they prevent rework and add value for patients, we did exclude them because their direct contribution is to improve safety. Our selection of tools was also based upon the literature used to select the business approaches (see Additional file 1: survey). The experts’ input and the pilot study were used to refine the list of tools. Altogether, 25 tools and groups of tools were selected. Additional file 1 describes the tools in more detail.

4. Goal accomplishment. We examined goal accomplishment on three performance aspects from an organizational perspective: efficiency, throughput times and financial results. Efficiency was defined as the relative number of inputs needed to achieve the intended outputs. The throughput time consisted of the total time needed to deliver a service from the moment demand is raised. Financial results were defined as the amount of money saved or earned as a result of the improved patient logistics. Yasin et al. [18] and Alexander et al. [20] suggested the use of multiple performance aspects in hospitals. Respondents scored the performance achieved on each aspect on a qualitative scale with six answers: 1) results had exceeded the goals, 2) goals had been accomplished, 3) goals had not been accomplished, but performance improved, 4) goals had not been accomplished, no change with regard to the performance aspect 5) goals had not been accomplished and performance decreased, 6) unknown.

5. Use of external support on patient logistics and internal training programs. Four questions asked whether an external organization had been involved 1) to analyse processes, 2) to support programs, 3) to implement changes or 4) to educate employees. Three answers were possible: No, external commercial organization, or external research organization. Questions were also asked on the availability of internal training programs for 1) management, 2) medical professionals and 3) supportive staff. Possible answers were: one-time education session, permanent education or knowledge sharing program, no training program available.

6. Evaluation methods. To examine the strength of evidence of the results, respondents were asked to describe the type of evaluation (quantitative, qualitative) and the method of data gathering (sample, pre-post measurement). Lastly, we asked whether the hospital had published on their improvement efforts.

For this research no new data on human subjects (including human material or human data) were collected, therefore the study design and the survey were not submitted to an ethics committee. This is in accordance with Dutch ethical guidelines.

### Sample

Staff advisors or managers responsible for patient logistics in Dutch general, academic, and non-academic teaching hospitals were asked to participate. The respondents were selected based on actual position enabling them to have an overview of patient logistics in their hospital and/or their registered or verifiable knowledge regarding patient logistics. We approached them through logistic networks for healthcare, personal contacts and hospital information. They received an invitation and reminder by e-mail, and, if necessary, a final reminder by telephone. Altogether, we approached staff in all 94 Dutch hospitals: eight academic teaching hospitals, 59 general hospitals, and 27 non-academic teaching hospitals [21].

### Data analysis

If at least, the questions on the hospital type and the approaches had been answered, surveys were included for analysis. We used mainly non-parametric data and descriptive statistics in the form of cross tables to analyse the data because the maximum sample size was 94 and the questions provided options for answers. In the cross tables we included only those hospitals that had provided answers on all questions relevant for that cross table, each table therefore shows the number of hospitals that has been included.

## Results

Of the 52 hospitals that returned the survey, six were excluded due to missing data, thus representing a response rate of 48% (n = 46). Table 1 presents the response rate per hospital type and the total number of hospitals of that type in the Netherlands.

**Table 1:** Type of hospital in survey relative to the total population in the Netherlands

***Type of hospital***	***Excluded***	***Included*** ***in research***	***Total number of hospitals*** ***in the Netherlands***	***Percentage of hospitals*** ***included in sample***
Academic teaching hospital	0	6	8	75
General hospital	4	27	59	46
Non-academic teaching hospital	2	13	27	48
Total	6	46	94	48

Table 2 shows that the 46 hospitals used care pathways most (91%), followed by benchmarking (78%). Focused factories (22%), lean six sigma (17%) and six sigma (13%) were used less frequently. Table 2 also shows the combinations of approaches used by hospitals. For example, 32 of the 42 hospitals that used care pathways (91%) also used benchmarking, 20 of the 42 used business process re-engineering and 20 used lean management. During the past two years, 98% had used multiple approaches, and 39% had used five or even more.

**Table 2:** Frequency of approaches used, and frequency with which they were combined (n = 46)

***Approaches***	**Total (%)**	CP*	BM*	BPR*	LM*	TOC*	CI*	TQM*	OR*	FF*	LSS*	SS*
Clinical pathways (CP)**	91		32	20	20	18	15	10	12	10	8	6
Benchmarking (BM)**	78	32		20	18	18	13	11	11	7	6	4
Business Process reengineering (BPR)**	48	20	20		14	10	6	6	10	6	5	4
Lean management (LM)**	48	20	18	14		13	7	4	8	7	2	5
Theory of constraints (ToC)**	43	18	18	10	13		8	6	7	6	2	4
Continuous improvement (CI)**	33	15	13	6	7	8		4	1	5	2	1
Total Quality Management (TQM)**	28	10	11	6	4	6	4		5	2	2	1
*Operations research (OR)* **	28	12	11	10	8	7	1	5		4	3	3
Focused factories (FF)**	22	10	7	6	7	6	5	2	4		1	1
Lean six sigma (LSS)**	17	8	6	5	2	2	2	2	3	1		3
Six sigma (SS)**	13	6	4	4	5	4	1	1	3	1	3	

* = Other approaches used by hospitals that used the approach indicated in the left-hand column. Result in column CP till SS in numbers.

** = approach used.

Regarding the approach that had been used most intensively over the previous two years, 26% of the hospitals (n = 46) reported multiple preferred approaches, and 13% did not prioritize a specific approach. Table 3 presents the most intensively used approaches per hospital type. Again, care pathways were used by far the most (35%), but business process re-engineering and lean six sigma (both 11%) received more mentions than benchmarking (9%). Lean management and total quality management were used in 9% of the hospitals.

**Table 3:** Most intensively used approaches per hospital type (N = 46)

**Approaches for patient** **logistics**	***Academic teaching*** ***hospitals (number)***	***General hospitals*** ***(number)***	***Non-academic teaching*** ***hospitals (number)***	***Total (%)***
Care pathways	4	9	7	35
Business process re-engineering	1	2	2	11
Lean six sigma	1	4	1	11
Benchmarking	0	5	0	9
Lean Management	0	4	0	9
Total Quality Management	1	1	1	9
Theory of Constraints	0	5	0	7
Collaborative improvements	0	1	0	2
Operations research	1	0	0	2
Focused factories	1	3	2	2
Six sigma	0	0	1	2

NB Multiple answers per hospitals were possible.

Table 4 shows which tools the hospitals used to improve patient logistics. Flowcharts were the commonest tool, of the 38 hospitals that answered this question: 16 used them “always”, 19 used them ”regularly”, and three used them “sometimes”. Standardized care pathways, elimination of waste, line balancing and identifying the capacity of the bottleneck were used always or regularly by at least 50% of the hospitals. The least frequently used tools scored less than 30% on the combination of “always” and “regularly”. They included quantitative tools such as DEA analysis, drum-buffer-rope principals, critical path analysis, and operations research techniques. Among these least frequently used tools were also tools such as process and outcome comparison that required collaboration with other organizations. Also 5 S, which concerns equipment and material storage and maintenance of the equipment and material, was not frequently used.

**Table 4:** The frequency of used tools or activities to improve patient logistics (in numbers)

**Tools**	**Total N**	**Always used**	**Regularly used**	**Sometimes used**	**Rarely used**	**Never used**
Use of flow charts	38	16	19	3	0	0
Standardized care pathways	36	5	22	6	3	0
Elimination of waste	37	5	12	10	7	3
Distinction between flow charts and value stream	34	5	5	12	4	8
Line balancing	35	4	20	7	3	1
Bottleneck has been identified	36	4	13	14	4	1
Cause-and-effect relations	38	4	8	12	10	4
Process time variability	35	2	19	11	3	0
Bottleneck has been quantitatively determined	34	2	14	11	6	1
Decide after quantifying effects	36	2	8	14	10	2
Reduce care demand variability	35	1	20	5	7	2
Reduce variability in capacity	35	1	18	11	5	0
Focus on patient group or service	34	1	11	11	5	6
Specific resources for focus groups	35	1	9	14	8	3
Variability pooling	32	1	7	5	10	9
Identifying best-practices together	35	1	4	11	13	6
Use of control charts	30	1	3	4	11	11
Simulation	32	1	2	3	12	14
Comparison of processes	34	0	5	13	12	4
Comparison of outcomes and inputs	33	0	4	13	11	5
Other operations research techniques than simulations	32	0	4	5	8	15
Use of 5 S	33	0	3	7	10	13
Critical path analysis	32	0	3	3	7	19
Drum-buffer rope principals	29	0	3	1	3	22
DEA analysis	27	0	2	1	4	20

N = number of hospitals.

Table 5 shows per hospital type whether the goals of the improvement approach had been accomplished with regard to their different performance aspects (n = 35). Efficiency was evaluated in 89% of the hospitals, throughput times in 83%, and financial results in 74%. Forty-nine percent of the hospitals reported having accomplished their efficiency goals, 40% having achieved desired throughput times, and 40% having achieved the desired financial results. With regard to performance, general hospitals reported more accomplishments than failures: 12 having accomplished efficiency goals against six efficiency failures (a success rate of 67%), ten having achieved desired throughput times against six failures (63% success rate), ten successes regarding financial results against five failures regarding financial results (success rate: 67%). Academic hospitals scored more successes than failures only with regard to financial results, while non-academic teaching hospitals scored more failures than successes on all aspects.

**Table 5:** Goal accomplisment per performance aspect per type of hospital

	**Academic teaching** **hospitals (number)**	**Non academic teaching** **hospitals (number)**	**General hospitals** **number)**	**Total percentage** **(n = 35 per performance aspect) (%)**
Efficiency goals +	1	4	12	49
Efficiency goals -	3	5	6	40
Efficiency goals NE	1	1	2	11
Throughput times +	0	4	10	40
Throughput times -	4	5	6	43
Throughput times NE	1	1	1	17
Financial results +	2	2	10	40
Financial results -	1	6	5	34
Financial results NE	2	2	5	26

+ = goals have been accomplished or exceeded.

- = goals have not been achieved.

NE = goals have not been evaluated.

Table 6 shows the results per aspect of goal accomplishment per approach. Between one and 18 hospitals applied a specific approach. Due to the small number of respondents per approach, we could not conclude that one approach was more successful than the others. Overall, about 50% of the hospitals reported that they had accomplished their goals. For the most frequently prioritized approach – clinical pathways in 18 hospitals – eight hospitals reported having accomplished their goals with regard to efficiency, seven accomplished their goals with regard to throughput times, and seven accomplished their goals with regard to financial results. Lean management (n = 7) was the approach that was least often accomplished, the failures outperformed the success on all performance aspects.

**Table 6:** Goal accomplishment on each performance aspect per prioritized approach in numbers (n = 35)

	**CP**	**LM**	**LSS**	**BPR**	**TQM**	**BM**	**TOC**	**CI**	**OR**	**FF**	**SS**
Total responses per approach	18	7	6	4	3	2	2	2	1	1	1
Efficiency +	8	3	3	3	1	2	1	1	1	0	1
Efficiency -	8	4	3	1	1	1	1	1	0	1	0
Efficiency NE	2	0	0	0	1	1	0	0	0	0	0
Throughput times +	7	3	3	3	1	0	0	1	1	0	1
Throughput times -	8	4	2	1	1	1	0	1	0	1	0
Throughput times NE	3	0	1	0	1	1	1	0	0	0	0
Financial results +	7	2	3	1	2	0	1	1	0	0	1
Financial results -	6	4	1	1	0	1	0	0	0	0	0
Financial results NE	5	1	2	2	1	0	1	1	1	1	0

N = total number of responses.

+ = goals have been accomplished or exceeded.

− = goals have not been accomplished.

NE = goals have not been evaluated.

The question on the use of external support to improve patient logistics was answered by 37 hospitals (see Table 7). Most hospitals used external support for analysis and education (both 68%). To support improvement programs 54% of the hospitals used external support and for the implementation of changes only 35%. The hospitals preferred external support from commercial organizations to that of research organizations on all aspects. The second part of Table 7 shows that permanent training programs on patient logistics were relatively scarce: 24% had a permanent program for management and supportive staff, 14% for medical professionals.

**Table 7:** Results on use of external support and internal training programs

***Use of external support to improve patient logistics***	**External commercial organization**	**External research organization**	**No external support used**	**Total n**
Analysis	20 (54%)	5 (14%)	12 (32%)	37
Support of programs	17 (46%)	3 (8%)	17 (46%)	37
Implementation of changes	10 (27%)	3 (8%)	24 (65%)	37
Education/training	20 (54%)	5 (14%)	12 (32%)	37
***Staff education/training programs***	**One-time education/training program**	**Permanent education/training program**	**No education/training program**	**Total n**
Management	17 (46%)	9 (24%)	11 (30%)	37
Medical professionals	12 (32%)	5 (14%)	20 (54%)	37
Supportive staff	12 (32%)	9 (24%)	16 (43%)	37

Between 32 and 36 hospitals provided information on their evaluation methods (for more details see Additional file 2). Eighty-six percent of the hospitals performed a quantitative evaluation, and 92% a qualitative evaluation. Eighty-nine percent of the hospitals that performed an evaluation performed a baseline measurement, 59% measured the results during the implementation period, and 53% performed a post intervention measurement. Forty-six percent of the hospitals repeated their periodic samples and 50% continuously measured their performance indicators. Only 14 hospitals published their results externally, four did this in a scientific journal.

## Discussion

This study presented the results of a survey on 1) the use of approaches to improve patient logistics in Dutch hospitals, 2) the tools used to support these approaches, 3) the reported effects of these approaches on performance, 4) the support structure that was applied, 5) and the methods used to evaluate the effects of the approaches on performance. The overall response rate to the survey was 48%, our sample contained 75% of the academic hospitals, 48% of the non-academic teaching hospitals, and 46% of the general hospitals. The response rate is therefore representative [22].

As 98% of the hospitals in the survey used different approaches and 26% prioritized multiple methods, most hospitals appeared not to have selected a single improvement approach. One possible reason for this is that hospitals allow various kinds of bottom-up initiatives, either because staff may have (random) knowledge on specific approaches or the selection of an approach may have been influenced by external agencies, or because there is too little evidence on the effectiveness of particular approaches. One reason given in literature is that hospitals may feel coercive, mimetic or normative pressures [23] that compel them to apply specific approaches because proponents of these approaches claim that performance will be improved. Both of these explanations should be investigated in future research.

The tools most frequently used are flow charts, standardized care pathways, waste elimination, distinction between flow charts and value, and line balancing. This may be because these tools do not seem to require much specific training or previous knowledge, are applicable to a wide range of settings, and belong to various improvement approaches (including CP, LM, LSS, SS and BPR). Denis et.al. and Rogers have shown that if an innovation – such as a tool for patient logistics – is perceived as simple, it is adopted more easily than a complex innovation [24,25]. This could explain the limited use of tools requiring specific operations management expertise, such as DEA analysis and the critical path method. The limited use of comparative methods – whereby inputs and outcomes could be prepared and best practices be identified – might be explained by a reluctance to collaborate with other hospitals. 5 S might be used infrequently because it seems most suitable for material intensive processes such as the management of operating room equipment. However, not all improvement activities –for example, reducing the access time to a consultations department- require changes in the storage and maintenance of equipment and material.

Patient logistics appeared to be a rather new topic for most hospitals as 68% used external support on analysis and education. This conclusion was confirmed by the response on internal training programs; only 24% of the hospitals had a structural program.

Roughly 50% of the 35 hospitals reported that they had accomplished their goals with regard to efficiency, throughput times and financial results. This means that around 50% of the resources spent on implementing changes did not result in goal accomplishment. An explanation for this success rate may be that hospitals did not apply the approaches properly or they did not define achievable targets. Apart from investing in knowledge on the approaches and implementation skills, increasing their focus on performance measurement and benchmarking might be helpful in improving the goal accomplishment rate.

To our knowledge, research on the effects of approaches to improve patient logistics in hospitals is often restricted to case studies within a single organization. Although publication bias is rather likely to occur, the reported goals accomplishment rate seems relatively low when we consider the reported claims made in most case studies. For references to cases see literature reviews [10-13,15,17,26]. There might thus be a publication bias towards success stories. The success rate also seems in contrast with that of Yasin et al. [18], who rated the effectiveness of approaches in US hospitals at between 68.2% (BPR) and 100% (CI). Further research should explain why goals have not been accomplished and whether or not there are any differences between countries.

General hospitals reported a higher degree of goal accomplishment than the academic hospitals and the non-academic teaching hospitals. One explanation could be that general hospitals are smaller and less complex, and therefore it is less difficult to create an environment that supports healthcare quality improvement [24,27]. Another reason lies in the positive association between experience with improvement approaches and the results achieved [28-30]. As general hospitals have been exposed to a competitive environment for a longer period, they may have more experience in working on patient logistics. Both reasons for the difference between the hospital types require further research.

With regard to the evaluation methods, 44% of the hospitals used a post-intervention measurement. Other research reported even lower evaluation rates: 19% in a literature review on lean management and six sigma [13]. As only four hospitals had published their findings scientifically, the improvement inputs covered in this study have so far produced only limited scientific evidence. This confirmed our hypothesis that the literature did not provide a complete picture of improvement efforts and their outcomes. This bias in literature would be logically overcome by the publication of more results, but this might be difficult to achieve in practice, as publication might eventually reduce a hospital’s competitive advantage.

Partly due to the low number of cases, we could not find any significant leads to establish a relationship between specific patient logistic approaches and goal accomplishment (see Table 6). While this might also be explained by the inappropriate use of approaches or by ineffective approaches, another possibility lies in the existence of contingency factors. Sousa and Voss suggest that contingency factors can affect the outcomes of operations-management interventions [31]. This makes the existence of a universal best practice for all organizations unlikely. The contingency factors affecting the outcomes of interventions in healthcare have been studied very little [32]. To determine which approach works best in a specific setting, future research should examine the effectiveness of the approaches and the contingency factors that apply to them.

### Research limitations

While we believe that our sample was representative, it may have been subjected to respondent bias. Two hospitals that did not respond indicated that they were focusing on the introduction of an electronic patient file system and a safety management system. The non-responding hospitals might have reported lower use of approaches and tools.

Like Yasin et al. [18], we approached one respondent per hospital. This may have led to single-source measurement bias. We deliberately selected our respondents based on their expertise on patient logistics and their knowledge of the hospital setting. In future research the dependency on respondents’ perceptions could be reduced by using valid measurement instruments to determine the use of a specific approach or tool and its effect on a hospital’s performance. Currently, however, very few validated instruments are available. There are few performance indicators, and even fewer are likely to reflect the direct relationship between improvement efforts and performance. The information needed to develop such instruments can be generated by in-depth case studies that combine literal and theoretical replication [33]. The alternative of multiple respondents per hospital can be used, but it remains questionable whether sufficient knowledgeable respondents can be identified.

## Conclusions

Patient logistics appeared to be an emerging topic for most hospitals as they relied on external support and did not have internal training programs. Nearly all hospitals (98%) used multiple approaches to improve patient logistics. Was this a decisions made by management of had management allowed bottom-up strategies? Overall, only 50% of the hospitals indicated that the approaches had enabled them to accomplish their goals with regard to patient logistics. No single approach seemed to outperform the others. It is unclear whether the use of multiple approaches or the available knowledge on the approaches affected the goal accomplishment rate. To generate the necessary understanding to make improvement efforts on patient logistics more successful, hospitals should participate in research on the selection and application of approaches, contingency factors and goal-setting procedures. They should also exchange positive and negative experiences on the approaches, and consider using more rigorous evaluation methods. Until better information becomes available, hospitals selecting an approach or combination of approaches will be left to rely mainly on their own experience and judgment.

## Competing interests

All authors worked for one of the hospitals that responded to the survey.

## Authors’ contribution

Wvl participated in the study design, the data collection and data analysis. She also drafted the manuscript. ES developed the study design and participated in data collection and data analysis. Furthermore, she contributed to the revision of the manuscript. WvH participated in the study design and contributed to the intellectual content of the manuscript. All authors read and approved the final manuscript.

## Pre-publication history

The pre-publication history for this paper can be accessed here:

http://www.biomedcentral.com/1472-6963/12/232/prepub

## Supplementary Material

Additional file 1 Additional file Survey translated to English. Click here for file

Additional file 2 Additional data Table A Evaluation characteristics.doc, 30 K Click here for file
