# Supplementary material for: Exploring improvements in patient logistics in Dutch hospitals with a survey
Source: BMC Health Serv Res. 2012 Aug 1;12:232. doi: 10.1186/1472-6963-12-232 (PMC3496592; doi:10.1186/1472-6963-12-232)
Supplement: Additional file 1 — Additional file Survey translated to English. [file 1472-6963-12-232-S1.docx]

# Section 1: Hospital type

1. What is the name of the hospital you are working for?

*This information was used to determine the hospital type: general hospital, non-academic teaching hospitals (not affiliated to universities), and academic teaching hospitals (affiliated to universities).*

# Section 2: Applied business approaches

1. *How would you characterize the use of business approaches to improve patient logistics in your hospital for the past 2 years? Please indicate in the following table which business approaches have been used in your hospitals the past 2 years (multiple answers possible). Please provide also the approach that has been used most intensively (1 answer possible)*

| Approaches to patient logistics (with abbreviation) | Applications in healthcare literature *This columns was not included in the survey* | Definition | Approach has been used | Most intensively used approach |
| --- | --- | --- | --- | --- |
| Total Quality Management (TQM) | [1] | “TQM is a method for ridding people’s lives of wasted effort by involving everyone in the processes of improvement; improving the effectiveness of work so that the results are achieved in less time. The methods and techniques used in TQM can be applied throughout the organization. They are equally useful to finance, sales, marketing, distribution, development, manufacturing, public relations, personnel, to every one of a company’s activities “ [2] |  |  |
| Business Process  Re-engineering (BPR) | [3] | “The fundamental rethinking and radical redesign of business processes to achieve dramatic improvements in critical, contemporary measures of performance, such as cost, quality, service, and speed “ [4] |  |  |
| Operations Research (OR) | [5] [6;7] | “Operations research is a scientific approach of providing executive departments with a quantitative basis for decisions regarding the operations under their control” [8] |  |  |
| Lean Management (LM) | [9]  [10] [11] | “An integrated socio-technical system whose main objective is to eliminate waste by concurrently reducing or minimizing supplier, customer and internal variability” [12] |  |  |
| Six Sigma (SS) | [13;14] | “Six Sigma methodology provides the techniques and tools to improve the capability and reduce the defects in any process. It improves any existing business process by constantly reviewing and re-tuning the process” [15] |  |  |
| Lean six Sigma (LSS) | [15;16] [17] | “A combination of lean management and six sigma. It is a methodology that maximizes shareholder value by achieving the fastest rate of improvement in customer satisfaction, cost, quality, process speed, and invested capital”[17] |  |  |
| Theory of Constraints (TOC) | [18] [19] [20;21] | “A [management](http://www.accountingformanagement.com/management_definition.htm) approach that emphasizes the importance of managing constraints. A [constraint](http://www.accountingformanagement.com/constraint_definition.htm) or [bottleneck](http://www.accountingformanagement.com/bottleneckneck_definition.htm) is any thing that prevents you from getting more of what you want.” [22] |  |  |
| Care Pathways (CP) | [23-25] | “This are structured multidisciplinary care plans which detail essential steps in the care of patients with a specific clinical problem.”[23] |  |  |
| Benchmarking (BM) | [26] [27] [28] [29] | “The search for- and implementation of best practices”.[27] |  |  |
| Collaborative Improvement (CI) | [30-32] | “A collaborative brings together groups of practitioners from different healthcare organizations to work in a structured way to improve one aspect of the quality of their service”. [33] |  |  |
| Focused Factories (FF) | [34-37] | “Its entire apparatus is focused on accomplishing the particular manufacturing task demanded by the company’s overall strategy and marketing perspective” [38] |  |  |
| Other |  |  |  |  |

**Answer the following questions based on your answer for the business approach that has been used most intensively for the past 2 years.**

# Section 3: applied tools

1. Please indicate in the following table the frequency with which your hospital applied tools or activities related to a specific approach to improve patient logistics.

|  |  | Intensity of tools used/number of hospitals | | | | |
| --- | --- | --- | --- | --- | --- | --- |
| Tools Not provided in the survey | Description | Always | Regularly | Sometimes | Rarely | Never |
| Use of flow charts / process schemes | Processes are described in flow charts or flow charts |  |  |  |  |  |
| Distinction between flow chart and value stream | Distinction between flow chart and value stream is made. |  |  |  |  |  |
| Capacity variability | Focuses on reduction of variability in available capacity |  |  |  |  |  |
| Cause and effects relations | Tools used to analyze cause and effect relations such as Ishikawa diagrams, cause & effects diagrams, 5 why. |  |  |  |  |  |
| Use of 5S | 5S is used, tool to organize the workplace: sort, set in order, standardize, and sustain. |  |  |  |  |  |
| Care demand variability | Control variability in demand (forecasting) |  |  |  |  |  |
| Process time variability | Reduce variability in the duration of an activity (diagnostics/treatment), also called process time variability. |  |  |  |  |  |
| Standardized care pathways | Standardize the different activities undertaken during diagnosis and/or treatment. |  |  |  |  |  |
| Use of control charts | Control charts are used to check whether variation is acceptable. |  |  |  |  |  |
| Elimination of waste | Non value adding activities are eliminated |  |  |  |  |  |
| Bottleneck has been identified | Bottleneck and bottleneck capacity have been identified |  |  |  |  |  |
| Line balancing | To balance the capacity of all activities that together make up the process. |  |  |  |  |  |
| Bottleneck has been quantitatively determined | The bottleneck can be quantitatively determined |  |  |  |  |  |
| Critical path analysis | The critical path for a series of activities is calculated (PERT/CPM/critical path analysis) |  |  |  |  |  |
| Drum-Buffer-Rope principal | Drum-Buffer-Rope principles are used to identify the bottleneck and to solve problems |  |  |  |  |  |
| Other operations research techniques than simulations | The following modelling techniques are used: decision trees, multivariate analysis, optimisation techniques, petri nets, queuing theory, survival analysis |  |  |  |  |  |
| Decision after quantifying the likely effects of changes | Consequences of changes are calculated/estimated before a decision is made. |  |  |  |  |  |
| Simulation | One of the following simulation types is used: agent-based simulation, discrete event simulation, gaming simulation, hybrid simulation, inverse simulation, monte carlo simulation, real time simulation, system dynamics |  |  |  |  |  |
| Comparing outcomes and inputs | Inputs and outputs of are compared with those of other organizations to identify improvement opportunities. |  |  |  |  |  |
| Comparing processes | Processes are compared with those of other organizations to identify improvement opportunities. |  |  |  |  |  |
| Identifying best practices together | Different professionals meet and use their expertise to decide what the best practices are |  |  |  |  |  |
| Data envelopment analysis (DEA analysis) | Data envelopment analysis is used to produce insight into multi-input, multi-output production functions by showing a production frontier. Often used to compare organizations with each other. |  |  |  |  |  |
| Focus on patient group or service | Hospital made a decision to serve a specific patient group and/or to deliver specific services. |  |  |  |  |  |
| Specific resources for focus groups | Specific resources are reserved for the patients on which the hospitals focuses. |  |  |  |  |  |
| Variability pooling | To reduce variability, similar treatments are planned in series (batch processing). This creates various savings, such as shorter changeover times between surgeries. |  |  |  |  |  |

# Section 4: goal accomplishment

1. Please indicate the rate of goal accomplishment of the most intensively used business approach on the following performance aspects

|  | Goals achieved | | Goals not achieved | | | Not evaluated |
| --- | --- | --- | --- | --- | --- | --- |
| Performance objectives | Results had exceeded the goals | Goals had been accomplished | Goals had not been achieved, but performance improved | Goals had not been achieved, no change with regard to the performance aspect | Goals had not been achieved, and performance has decreased | Not evaluated |
| Efficiency |  |  |  |  |  |  |
| Throughput times |  |  |  |  |  |  |
| Financial performance |  |  |  |  |  |  |

# Section 5: Use of external support on patient logistics and internal training programs

1. Please indicate in the following table whether external organizations have been hired to support improvements on patient logistics with the most intensively used business approach.

| Organization has been hired to: | No external organization has been hired | External commercial organization | External research organization |
| --- | --- | --- | --- |
| Analyze processes |  |  |  |
| Support programs |  |  |  |
| Implement changes |  |  |  |
| Educate employees |  |  |  |

1. Please indicate in the following table whether training programs on patient logistics were available for the following target groups:

| Target group | One-time education session | Permanent education or knowledge sharing program | No training program available |
| --- | --- | --- | --- |
| Management |  |  |  |
| Medical professionals |  |  |  |
| Supportive staff |  |  |  |

# Section 6: Evaluation methods

1. For the evaluation of the most intensively used business approach, did the organization perform the following types of evaluation:
   1. Qualitative evaluation
   2. Quantitative evaluation
   3. Baseline measurement
   4. Measured the results during the implementation
   5. A single post implementation measurement
   6. Periodic samples
   7. Measured continuously
2. Have the results been published?
   1. Internally
   2. External scientific publication, where was it published?
   3. External non scientific publication, where was it published?
   4. Not published

Reference List

[1] Õvretveit J: **Total quality management in European healthcare.** *Int J Health Care Qual Ass* 2000;**13**(2):74-80.

[2] John S Oakland. Total Quality Management. London: Butterworth-Heinemann Ltd, 1989.

[3] Elkhuizen SG, Limburg M, Bakker PJM, Klazinga NS: **Evidence-based re-engineering: re-engineering the evidence: A systematic review of the literature on business process redesign (BPR) in hospital care.** *Int J Health Care Qual Ass* 2006;**19**(6):477-499.

[4] Hammer M, Champy J. Business process re-engineering. London: Nicholas Brealey 1993.

[5] Research Into Global Healthcare Tools (RIGHT). Modelling and Simulation Techniques for Healthcare Decision Making: A Selection framework. Cambridge, UK: Engineering Design Centre, University of Cambridge, 2008.

[6] Brailsford SC, Harper PR, Patel B, Pitt M: **An analysis of the academic literature on simulation and modelling in health care.** *Journal of Simulation* 2009;**3**(3):130-140.

[7] Van Oostrum JM: Applying mathematical Models to Surgical Planning. Erasmus Research Institute of Management - ERIM PhD Series in Research in Management, 2009.

[8] Morse PM, Kimball GE. Methods of Operations Research. New York: Dover Publications, 2003.

[9] Langabeer JR, DelliFraine JL, Heineke J, Abbass I: **Implementation of Lean and Six Sigma quality initiatives in hospitals: A goal theoretic perspective.** *Operations Management Research* 2009;**2**(1):13-27.

[10] Mazzocato P, Savage C, Brommels M, Aronsson H, Thor J: **Lean thinking in healthcare: a realist review of the literature.** *Qual Saf Health Care* 2010. Epub 2010 Aug 19.

[11] Benders J, Santbergen S: **'Lean'in Nederlandse ziekenhuizen: Een overzicht van eerste ervaringen.** *M&O, Tijdschrift voor Management en Organisatie* 2007;**61**(2):36-47.

[12] Shah R, Ward PT: **Defining and developing measures of lean production.** *Journal of Operations Management* 2007;**25**(4):785-805.

[13] van den Heuvel J, Does RJMM, Bogers A, Berg M: **Implementing six sigma in the Netherlands.** *Jt Comm J Qual Pat Saf* 2006; 32(7):393-399.

[14] Frings GW, Grant L: **Who moved my sigma effective implementation of the Six Sigma methodology to hospitals.** *Quality and Reliability Engineering International* 2005;**21**(3):311-328.

[15] de Koning H, Verver JPS, van den Heuvel J, Bisgaard S: **Does R. Lean six sigma in healthcare.** *J Healthc Qual* 2006;**28**(2):4-11.

[16] Heuvel JVD, Does RJMM, Koning HD: **Lean Six Sigma in a hospital.** *International Journal of Six Sigma and Competitive Advantage* 2006;**2**(4):377-388.

[17] George ML. Lean six sigma for service: How to use lean speed and six sigma quality to improve services and transactions. McGraw-Hill Professional, 2003.

[18] Breen AM, Burton-Houle T, Aron DC: **Applying the theory of constraints in health care: part 1-the philosophy.** *Qual Manag Health Care* 2002;**10**(3):40-46.

[19] Womack DE, Flowers S: **Improving system performance: a case study in the application of the theory of constraints.** *J Healthc Manag* 1999;**44**(5):397-405.

[20] Borghuis T.L., Bos W.J.W., Geers A.B., Steenhoff A.M.T., Muis G, de Wit M et al: **Vertragingen te lijf.** *Medisch Contact* 2007;33-34.

[21] Paree M.J.M., T.L.Borghuis-Lub: **Klinische paden en TOC: samen beter.** *Medisch Contact* 2010;(33/34).

[22] Goldratt Eliyahu M. Theory of Constraints. North-River Press, Croton-on-Hudson, NY 1990.

[23] Campbell H, Hotchkiss R, Bradshaw N, Porteous M: **Integrated care pathways.** *BMJ* 1998;**316**(7125):133-137.

[24] Sermeus W, Vanhaecht K, Vleugels A: **The Belgian-Dutch clinical pathway network.** *Journal of Integrated Care Pathways* 2001;**5**(1):10-14.

[25] Vanhaecht K, Bollmann M, Bower K, Gallagher C, Gardini A, Guezo J et al: **Prevalence and use of clinical pathways in 23 countries-an international survey by the European Pathway Association.** *Journal of Integrated Care Pathways* 2006;**10**(1):28.

[26] van Lent W, de Beer R, van Harten W: **International benchmarking of specialty hospitals. A series of case studies on comprehensive cancer centres.** *BMC Health Serv Res* 2010;**10**(1):253-264.

[27] Camp RC. Benchmarking: The Search for Industry Best Practices that Lead to Superior Performance. Milwaukee,(USA) 1989.

[28] Mosel D, Gift B: **Collaborative benchmarking in health care.** *Jt Comm J Qual Improv* 1994; 20(5):239-249.

[29] Borghans I, Heijink R, Kool T, Lagoe RJ, Westert GP: **Benchmarking and reducing length of stay in Dutch hospitals.** *BMC Health Serv Res* 2008;**8**(1):220.

[30] Dückers M, Makai P, Vos L, Groenewegen P, Wagner C: **Longitudinal analysis on the development of hospital quality management systems in the Netherlands.** *Intl J Qual Health* Care 2009;**21**(5):330-340.

[31] Schouten LMT, Hulscher MEJL, Everdingen JJE, Huijsman R, Grol RPTM: **Evidence for the impact of quality improvement collaboratives: systematic review.** BMJ 2008;**336**(7659):1491.

[32] Schouten LMT, Hulscher MEJL, Akkermans R, van Everdingen JJE, Grol RPTM, Huijsman R: **Factors that influence the stroke care team's effectiveness in reducing the length of hospital stay.** Stroke 2008;**39**(9):2515.

[33] Õvretveit J, Bate P, Cleary P, Cretin S, Gustafson D, McInnes K et al.: **Quality collaboratives: lessons from research.** *Qual Saf Health Care* 2002;**11**(4):345.

[34] Herzlinger R. What Works: Health Care Focused Factories. Market-Driven Health Care 1997.

[35] Herzlinger R: **Retooling healthcare.'Focused factory'model can help build a patient-friendly, service-driven system.** *Mod Healthc* 1997;**27**(7):96.

[36] Bredenhoff E, van Lent WAM, van Harten WH: **Exploring types of focused factories in hospital care: a multiple case study.** *BMC Health Serv Res* 2010;**10**(1):154-170.

[37] Pieters A, van Oirschot C, Akkermans H: **No cure for all evils: Dutch obstetric care and limits to the applicability of the focused factory concept in health care*.*** *International Journal of Operations & Production Management* 2010;**30**(11):1112-1139.

[38] Skinner W. The focused factory. Harvard Business Review 1974.
