# Supplementary material for: Exploring improvements in patient logistics in Dutch hospitals with a survey
Source: BMC Health Serv Res. 2012 Aug 1;12:232. doi: 10.1186/1472-6963-12-232 (PMC3496592; doi:10.1186/1472-6963-12-232)
Supplement: Additional file 2 — Additional data Table A Evaluation characteristics.doc, 30 K [file 1472-6963-12-232-S2.doc]

Table A Evaluation characteristics

| Evaluation characteristics | N | Application of characteristics |
| --- | --- | --- |
| Quantitative evaluation  Qualitative evaluation | 36 | 86%  92% |
| Baseline measurement | 35 | 94% |
| Measured the results during the implementation | 35 | 59% |
| Post measurement | 34 | 53% |
| Periodic samples | 32 | 46% |
| Measured continuously | 34 | 50% |
| Publication:   - External - Scientific publication | 35 | 41%  12% |
